# Late onset of hemolytic uremic syndrome after the appearance of prodromal gastrointestinal tract symptoms

**DOI:** 10.1002/ccr3.3020

**Published:** 2020-06-10

**Authors:** Chihiro Hirata, Tsuneaki Kenzaka, Hozuka Akita

**Affiliations:** ^1^ Department of Internal Medicine Hyogo Prefectural Tamba Medical Center Tamba Japan; ^2^ Division of Community Medicine and Career Development Kobe University Graduate School of Medicine Kobe Japan

**Keywords:** delayed onset, enterohemorrhagic *Escherichia coli*, hemolytic uremic syndrome, shiga toxin

## Abstract

Hemolytic uremic syndrome (HUS) may occur late after the onset and improvement of gastrointestinal tract symptoms. Clinicians need to carefully monitor for the onset of HUS, even if the patients have few symptoms.

## Graphical abstract

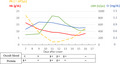

## 1 INTRODUCTION

A healthy 26‐year‐old female presented with enterohemorrhagic *Escherichia coli* with diarrhea for 8 days. On the 11th day after onset of illness, hemolytic uremic syndrome developed, even though she had few symptoms. Close follow‐up is needed even after improvement of gastrointestinal symptoms.

Hemolytic uremic syndrome (HUS) is a thrombotic microangiopathy induced by Shiga toxins that are produced by the enterohemorrhagic bacterium, *Escherichia coli*. The diagnostic criteria are: (a) hemolytic anemia [anemia with fragmented red blood cells (RBCs] and a hemoglobin (Hb) level of <10 g/dL); (b) thrombocytopenia (platelets <150 000/μL); and (c) acute renal injury (serum creatinine ≥1.5 times the normal limit).^1^

Enterohemorrhagic *Escherichia coli* (EHEC) is a diarrheal type of *E coli* that produces Shiga toxins and destroys epithelial cells in the large intestine, resulting in intestinal hemorrhage. All EHEC‐infected patients are duty‐bound by law to report a precedent to their respective public health centers. The number of patients reported in Japan is 3000‐4000 persons (2.5‐3.3 per 100 000 persons) per year.^2^ The incidence rate of EHEC infection is 0.6 per 100 000 persons in South Africa, 3.9 in Hong Kong, 12 in Chile, 30 in Bangladesh, and 5.9 in the United States, showing large regional differences.^3^, ^4^ Comparatively, the incidence in Japan is not particularly high.

The interval between ingestion of a contaminated vehicle and the onset of diarrhea ranges between 2 days and 12 days (median 3 days).^5^ Typically, the diarrhea eventually improves within 7 days.^5^ In general, HUS is observed in 1%‐10% of EHEC‐infected patients, and only approximately 10% of patients are adults while the majority are children.^1^ The onset of HUS usually occurs 4‐10 days after the onset of diarrhea or fever.^1^ Moreover, 40%‐70% of HUS cases are complicated by acute renal injury requiring dialysis, and 20%‐50% of patients present with central nervous system (CNS) symptoms.^6^, ^7^ CNS disorders, anemia, and thrombocytopenia tend to become severe and lead to an unfavorable prognosis, particularly in elderly patients.^1^

Herein, we report the case of an adult patient with delayed onset HUS preceded by gastrointestinal symptoms, presenting with few subjective symptoms, that was treated with conservative therapy alone.

## 2 CASE REPORT

A 26‐year‐old woman with a normal medical history presented with a complaint of watery stools.

She was a nonsmoker and an occasional drinker. The patient had consumed raw spring rolls at a restaurant 2 days prior. The patient developed a fever 2 days after the meal (Day 1 of onset of illness), excreted approximately 10 watery stools, and experienced lower abdominal pain starting on the next day. On Day 3 of the onset of illness, bloody stools and vomiting were observed; on Day 4, the patient underwent a check‐up at Hospital A. The patient was diagnosed with infectious gastroenteritis and was prescribed 500 mg/d of levofloxacin for 3 days. Although her bloody stools and vomiting improved, the patient could not eat and was still having a few watery stools per day. The patient was referred to our department on Day 7 of the onset of illness because EHEC O157 (positive for verotoxins type 1 and 2) was detected in the stool culture at Hospital A. The physical findings were as follows: height, 160 cm, and weight, 49 kg (no weight loss observed). Her vital signs were as follows: body temperature, 36.7°C; blood pressure, 114/74 mm Hg; pulse, 112 beats/min; respiratory rate, 16 breaths/min; and consciousness, clear. Dry mouth was not observed. The abdomen was flat and soft, with mild epigastric tenderness but no rebound tenderness. Intestinal peristalsis was reduced, and the capillary refill time was <2 seconds.

Her hematology and chemistry test findings are shown in Table 1. In the urinalysis, the specific gravity was 1.013, protein was 3+, occult blood was ±, ketone bodies were +, and erythrocytes in sediment were 30‐49/high power field.

**TABLE 1:** Laboratory data on admission

Parameter	Recorded value	Standard value
White blood cell count	13 580/µL	4500‐7500/µL
Neutrophils	76.0%	42‐74%
Lymphocytes	16.3%	18‐50%
Hemoglobin	16.4 g/dL	11.3‐15.2 g/dL
Hematocrit	46.4%	36%–45%
Platelets	219 × 10^3^/µL	130‐350 × 10^3^/µL
C‐reactive protein	1.64 mg/dL	≤0.60 mg/dL
Total protein	7.3 g/dL	6.9‐8.4 g/dL
Albumin	4.0 g/dL	3.9‐5.1 g/dL
Total bilirubin	0.9 mg/dL	0.2‐1.2 mg/dL
Direct bilirubin	0.3 mg/dL	0.0‐0.3 mg/dL
Aspartate aminotransferase	31 U/L	8‐38 U/L
Alanine aminotransferase	9 U/L	4‐44 U/L
Lactate dehydrogenase	301 U/L	120‐230 U/L
γ‐glutamyl transpeptidase	19 U/L	9‐32 U/L
Creatinine phosphokinase	54 U/L	40‐150 U/L
Blood nitrogen urea	13.4 mg/dL	8‐20 mg/dL
Creatinine	0.70 mg/dL	0.43‐0.75 mg/dL
Sodium	133 mEq/L	136‐148 mEq/L
Potassium	4.2 mEq/L	3.6‐5.0 mEq/L
Chloride	93 mEq/L	101‐108 mEq/L
Glucose	94 mg/dL	70‐109 mg/dL

**FIGURE 1:**
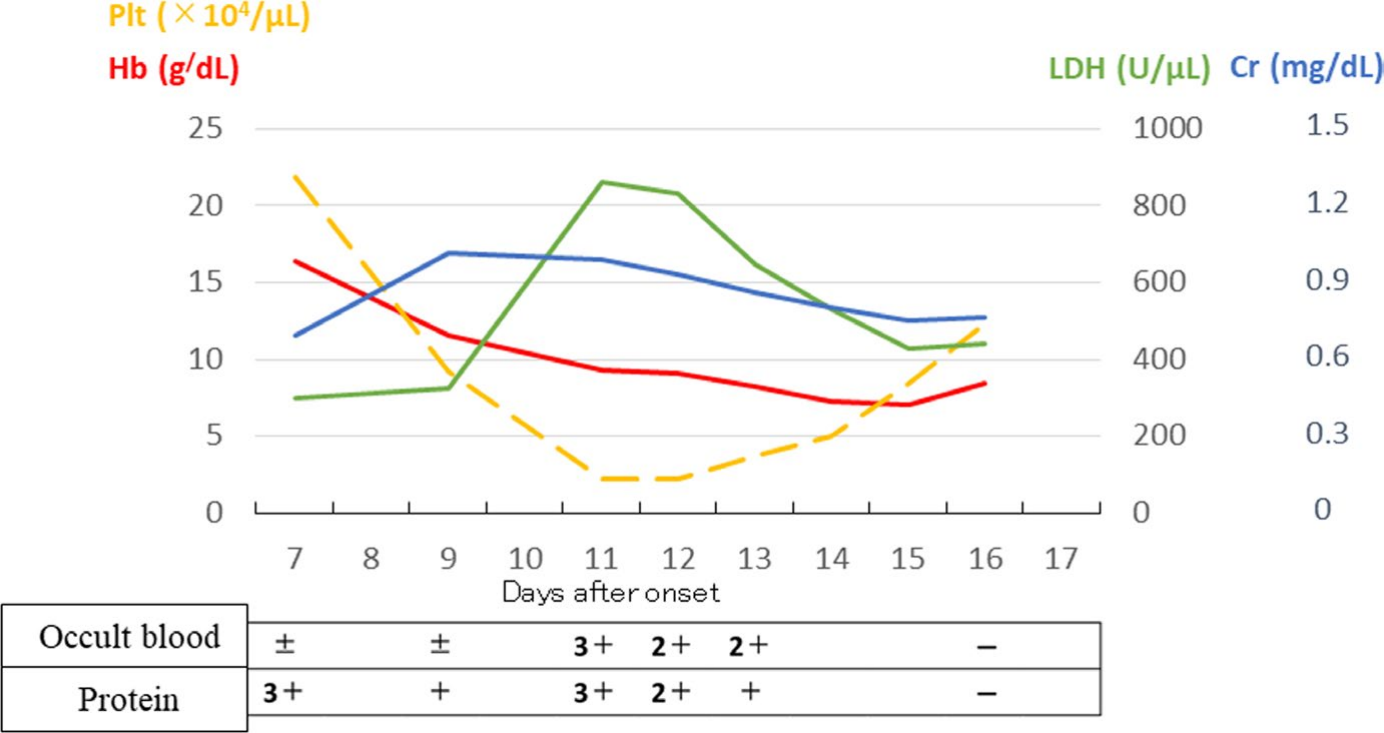
Changes in platelet counts, hemoglobin, lactate dehydrogenase, creatinine, and qualitative urinalysis findings

The patient's diarrhea had almost completely resolved at the time of admission; however, her food intake remained poor, and based on her positive urine ketones, we concluded that she was dehydrated. Thus, fluid therapy was administered during hospitalization as a symptomatic treatment. At the time of admission, measures to prevent contact infection (based on the hospital manual) were taken by the infection control team of the hospital. Initially, the dehydration was considered to be due to the protein 3+ and occult blood ± findings. The patient's diarrhea had completely resolved by Day 8 of the onset of illness. After admission, the patient had no subjective symptoms, appeared well, and was not critical. However, blood tests revealed thrombocytopenia, advanced anemia, an increase in lactate dehydrogenase (LDH) level, and a predominant increase in indirect bilirubin level over time. On Day 11 of the onset of illness, fragmented RBCs were observed, the LDH was 862 mg/dL, the total bilirubin level had increased to 3.9 mg/dL (direct bilirubin was 0.9 mg/dL), and the platelet count was the lowest at 22 000/μL. In addition, the direct Coombs test was negative, haptoglobin level was <10 mg/dL (n: 19‐170), and a disintegrin and metalloproteinase with a thrombospondin type 1 motif, member 13 (ADAMTS13) activity was 41% (normal range: >10). On the same day (Day 9), the urinary protein was 3+, the urinary occult blood worsened to 3+, and the serum creatinine (Cr) was 1.03 mg/dL, all of which indicated renal dysfunction. The three criteria for HUS, ie, hemolytic anemia, thrombocytopenia, and acute renal injury (which increased from 0.68 mg/dL to 1.03 mg/dL, 1.5‐fold from baseline) were met, and delayed onset HUS was diagnosed on Day 11. The platelet count was lowest on Day 11 and the Hb levels dropped to 7.0 g/dL on Day 15 as a result of the hemolytic anemia (Figure 1). An infusion of 2000‐3000 mL/d of extracellular fluid was started on Day 11, and the laboratory findings gradually improved (Figure 1). Although slight headache and nausea were observed for approximately 4 days beginning on Day 12, the patient's general condition was good and she was discharged on Day 17. Figure 1 shows the changes in platelet counts, Hb, LDH, Cr, and qualitative urinalysis findings.

On Day 26, during an outpatient consultation, it was confirmed that the patient was cured based on the improvement of her symptoms and positive laboratory test results, including hematology, chemistry, and urinalysis. The Cr value was 0.68 mg/dL. At a later date, the health center reported that two other people had been infected with O157 after eating at the same restaurant 2 days prior to the onset of the current patient's symptoms.

## 3 DISCUSSION

This report described a patient with delayed onset HUS that was preceded by gastrointestinal symptoms. In this patient, fragmented RBCs were first observed on Day 11 of the onset of illness, and HUS was diagnosed on the same day. In previous population studies of O157 infections, HUS has been shown to occur mostly on Days 5‐10 after the onset of diarrhea, and the time to diagnosis has been reported to be 6.5‐7.7 days after the onset.^8^, ^9^ A clinical study of 16 pediatric patients with HUS reported that abnormal laboratory values peaked at Day 7.0 ± 1.8 (mean ± standard deviation) after the onset of illness for thrombocytopenia, Day 7.5 ± 1.9 for an increase in LDH indicative of hemolytic anemia, and Day 7.2 ± 1.8 for decreased estimated glomerular filtration (eGFR). The peak values centered mainly around Day 7.2 ± 1.8 of the onset of illness.^10^ In our patient, the most abnormal laboratory values were observed on Day 11 for platelets and LDH (increase; an index of hemolytic anemia), on Day 15 for Hb (decrease), and on Day 9 for Cr (increase) and eGFR (decrease), demonstrating a delayed onset from the standard deviation of previous reports (except for acute renal injury). The typical symptoms of HUS are reported to be oliguria, edema, ecchymosis, headache, somnolence, restlessness, convulsion, and disturbed consciousness.^10^ A clinical study of 298 adult patients with HUS reported that abnormal laboratory values peaked at Days 6‐8 after the onset of illness for hemolytic anemia and thrombocytopenia and Day 8 for serum Cr^11^ The patient in this study, however, presented with few subjective symptoms, and the onset of HUS would not have been noticed if the patient had not been hospitalized.

A white blood cell (WBC) count ≥15 000/μL and a C‐reactive protein level ≥2.0 mg/dL are known risk factors for the progression of an EHEC infection to HUS.^12^ Meanwhile, a WBC count of ≥20 000/μL, Na < 130 mEq/L, total protein < 5.0 g/dL, and ALT >70 U/L, which are predictors of HUS severity, have been reported as risk factors for dialysis.^7^ Patients who develop HUS 2‐3 days after the onset of diarrhea tend to exhibit a rapid and serious disease course^1^; however, a contradictory study has reported that there is no relationship between the onset of HUS and the severity of enteritis symptoms, such as the presence or absence of hematogenous diarrhea and requirement of hospitalization.^13^ Although the patient in this study showed no abnormal laboratory values that met the aforementioned criteria and presented with slight symptoms, the onset of HUS and severe thrombocytopenia were identified.

The incidence of death in the acute phase of HUS has been reported to be 2%‐4%, with many CNS complications and gastrointestinal perforation.^14^, ^15^ However, some adults may have recovered spontaneously after an O157 infection, without being aware of the onset of HUS, as they may have exhibited a wide range of symptoms. As mentioned above, a study has reported that there is no relationship between the severity of symptoms and the onset of HUS. A significant risk factor for HUS is age <15 years or >65 years.^16^ Among adult patients with HUS. 20%‐40% show a gastrointestinal symptom, including bloody diarrhea, abdominal pain, vomiting, >10 stools/d, or abdominal tenderness.^16^ However, there is no significant difference in the occurrence of these symptoms between HUS patients and healthy adults.^11^ Owing to this contradictory information regarding infectious enteritis caused by EHEC, there is a need to prepare for the onset and deterioration of HUS by closely monitoring the changes in subjective symptoms and the results of hematology, chemistry, and urinalysis tests after symptoms of diarrhea have resolved, even if the symptoms do not appear to be severe.

It has been reported that administration of levofloxacin does not increase the frequency of HUS in O157‐infected patients.^17^ In addition, reports of autoimmune hemolytic anemia and thrombotic microangiopathy caused by levofloxacin are extremely rare,^18^, ^19^, ^20^ with all of them involving autoimmune hemolytic anemia and thrombotic microangiopathy during levofloxacin treatment.^18^, ^19^, ^20^ We believe that levofloxacin administration was not related to the HUS in this case.

In conclusion, this report described the case of a patient with late‐onset HUS that was preceded by the appearance of gastrointestinal symptoms, who was successfully treated with fluid therapy alone. In adults with an EHEC infection, HUS may occur after the improvement of gastrointestinal symptoms; therefore, careful monitoring of HUS symptoms and changes in hematology, chemistry, and urinalysis findings is required, regardless of the severity of symptoms. Our finding is important for improving the diagnostic accuracy of clinicians when dealing with gastrointestinal problems and supplementing their knowledge about the occurrence of HUS.

## CONFLICT OF INTEREST

The authors declare that they have no competing interests.

## AUTHOR CONTRIBUTION

CH: managed the case and redaction and corrected the manuscript. TK and HA: assisted with redaction, correction, and reconstruction of the manuscript. All authors read and approved the final manuscript.

## Data Availability

All data generated or analyzed during this study are included in this published article.
